# Informing tobacco control policy in Jordan: assessing the effectiveness of pictorial warning labels on cigarette packs

**DOI:** 10.1186/s12889-017-4642-8

**Published:** 2017-08-01

**Authors:** Rasha K. Bader, Rawan A. Shihab, Dalia H. Al-Rimawi, Feras I. Hawari

**Affiliations:** 1 King Hussein Cancer Center, Cancer Control Office, 202 Queen Rania Al Abdullah Street, POBox 1269, Amman, 11941 Jordan; 2 King Hussein Cancer Center, Research Office, 202 Queen Rania Al Abdullah Street, Amman, Jordan

**Keywords:** Pictorial warning labels, Assessment of effectiveness, Tobacco control policy

## Abstract

**Background:**

Pictorial warning labels (PWLs) deter initiation and motivate quitting. Assessing PWLs is important to track effectiveness and wear out. Jordan introduced an updated set of PWLs in 2013. This study assessed the effectiveness of the set after 2.5 years on the market.

**Methods:**

We administered a survey in a cross-sectional sample of young adults aged 17–26 years. For convenience, respondents were recruited on university campuses. For heterogeneity, respondents were solicited from the different schools in four geographically diverse university campuses. The study compared perceptions of effectiveness surveyed in 2015 to perceptions gauged in 2010 during a pre-launch evaluation exercise. Outcomes of interest were: salience, fear evocation, adding information, and ability to motivate quitting smoking (for smokers) or deterring starting (for non-smokers).

**Results:**

Results indicate awareness of the set among smokers and non-smokers, and their recall of at least one PWL message. Results also indicate effectiveness of the set: (1) 1/3 smokers who frequently saw them reported PWLs to trigger considering quitting, (2) and among both smokers and non-smokers the set in 2015 sustained ability to motivate quitting and staying smoke-free. However, results uncover erosion of salience, suggesting that the set has reached its end of life. Finally, results reveal variability in performance among PWLs; the one PWL that depicts human suffering significantly outperformed the others, and its ability to motivate was most strongly associated with its ability to evoke fear.

**Conclusion:**

Based on the early signs of wear-out (i.e. erosion of salience), and understanding the importance of sustaining upstream outcomes (especially fear evocation) to sustain motivation, we recommend retiring this set of PWLs and replacing it with a stronger set in line with proven standards.

**Electronic supplementary material:**

The online version of this article (doi:10.1186/s12889-017-4642-8) contains supplementary material, which is available to authorized users.

## Background

Tobacco use is linked to numerous diseases and is globally considered the leading cause of preventable death [1]. Recognizing this principle, the Framework Convention on Tobacco Control (FCTC) sets forth evidence-based strategies that, when implemented, help countries address the tobacco epidemic [2]. One of the most cost-effective demand-reduction strategies of the FCTC is introducing health warnings to the packaging of tobacco products with the purpose of communicating health risks and reducing tobacco use [2, 3].

Article 11 of the FCTC provides guidelines for effective warnings, including measures that enhance their prominence [2, 3]. Effective warnings should be large, clear, visible, and legible, and should utilize a set of rotating messages. Preferably, they should occupy the top part of the principal display areas (front and back of the packaging) and occupy 50% or more of those areas.

For enhanced effectiveness, Article 11 recommends the use of pictures or pictograms to form pictorial warning labels (PWLs), preferably through utilizing graphic or shocking images [2, 3]. Strong PWLs have been shown to dissuade from experimentation, deter initiation, motivate quitting or decreased consumption, and support those who quit in staying abstinent [2–8]. These effects give PWLs additional importance in low- and middle-income countries (LMICs) where mass media campaigns are lacking, thus rendering PWLs one of the few sources of information on the effects of smoking [9, 10].

Article 11 of the FCTC further details measures for sustained benefit, including conducting periodic reviews and assessments to address wear out and dilution of impact [3, 9, 11, 12]. While wear out could occur as early as one year after launching, [9, 12] stronger warnings tend to sustain their impact for longer periods of time [13].

Compared to countries of the Eastern Mediterranean Region (EMR) and other parts of the world, Jordan is heavily burdened with tobacco use. Prevalence among adult males exceeds 70%, [14] and 34% of boys (ages 13–15) currently use some form of tobacco products [15]. In line with the requirements of the FCTC to which it has been a party since 2004, [16] Jordan enforced one PWL in 2006 (covering 30% of the packet area) [17]. Regrettably, no structured pre-launch testing or post-launch assessment of this PWL was conducted [18].

Aiming to strengthen compliance with FCTC requirements, Jordan followed that single PWL with a replacement set of PWLs which was launched in early 2013 and continues to be in circulation today [17, 19]. The new set includes four rotating PWLs covering 40% of the lower back area of the packet (where 2/3 of the space is occupied by a picture and 1/3 by text, and a thick border is included within the space), along with a fixed text warning covering 40% of the lower front area (to view the set of PWLs visit http://global.tobaccofreekids.org/files/pdfs/en/WL_country_Jordan_en.pdf). While this set increased the area covered and the number of PWLs in circulation, it did not introduce other changes deemed critical to strengthen effectiveness; [2, 3, 20] namely moving the PWL to the upper front surface of the packet and increasing its size to 50% of the packet area. Accordingly, despite being one of the first countries to implement PWLs, [19] Jordan today does not score well on fulfilling FCTC requirements with regards to Article 11, and lags behind other countries in the region and the world including late adopters [19, 21–23].

This study sought to assess the effectiveness of the new set of PWLs after having them circulate on the market for 2.5 years. To do so, the study compared perceptions of the set in 2015 to the immediate perceptions of the same set as gauged in 2010 during a pre-launch evaluation exercise [18]. Building on this comparison, the study generated recommendations for strengthening the effectiveness of PWLs in Jordan and their compliance with the guidelines set by the FCTC.

## Methods

### Sample

In line with the design used in the pre-launch evaluation, [18] a cross-sectional sample of young adults (ages 17–26) was targeted for this long-term assessment. For convenience, respondents were recruited on university campuses. Out of 1250 survey copies delivered to the surveyors at the initiation of the data collection phase, a total of 1101 completed surveys were returned. After conducting data checks and data cleaning, responses from a final sample of 920 respondents were considered reliable for data analysis.

### Instrument

An adaptation of the instrument that was utilized during the pre-launch evaluation was used for this study [18]. The Arabic instrument consisted of four sections. The first section collected demographic information (sex, age, and education). The second section gauged the respondents’ opinions on the harms of smoking and on the need for PWLs. The third section assessed the respondents’ notice and recall of the PWLs in circulation and their general reactions to these PWLs. Finally, the fourth section gauged the respondents’ perceptions of each of the four PWLs in circulation through four identical subsections.

Each of the subsections of the fourth section addressed one of the PWLs in circulation. Upon prompting the respondent with an image of the pertinent PWL as a recall aid, each subsection started with a prequalifying question (Have you previously seen this warning?) which served as a filter based on which the respondent was requested to provide feedback. Those responding positively to this question, were requested to rate the PWL on four outcomes: salience, fear evocation, adding information, and ability to motivate quitting smoking (for smokers) or deterring starting (for non-smokers) [5, 7, 24]. A five-point Likert scale was used for all ratings, ranging from ‘1’ to ‘5’ or from ‘strongly disagree’ to ‘strongly agree’.

The survey instrument was reviewed and approved by the Institutional Review Board at King Hussein Cancer Center. An English translation is available electronically as Additional file 1.

### Procedures

In May 2015, 25 volunteers were recruited through Jordan’s chapter of the International Federation of Medical Students Association to serve as surveyors (volunteers received no remuneration but were compensated for transportation costs). Prior to embarking on data collection, the volunteers were introduced to the purpose and the design of the study; trained on the survey tool and interviewing methods; and trained on how to identify potential respondents in common areas, how to approach them to introduce the study and confirm that they fall within the targeted age group, and how to obtain verbal consent from those willing to participate prior to administering the survey.

Recruitment of respondents followed certain guidelines. To ensure geographic diversity, the volunteers came from four university campuses (*Jordan University of Science and Technology* in the northern region, *University of Jordan* and *Hashemite University* in the central region, and *Mutah University* in the southern region). To ensure diversity in the educational background of the respondents, each surveyor recruited respondents from the various schools on his/her own university campus. Surveyors approached potential respondents, explained the purpose and procedure for the study, highlighted that participation was voluntary and that the respondents could withdraw at any time, and highlighted that no identifying information would be collected. Once a respondent provided verbal consent, a surveyor spent up to 30 min interviewing the respondent and documenting responses in the survey tool. Respondents received no compensation.

### Measures and data analysis

Data analysis was conducted using SAS version 9.4 (SAS Institute Inc., Cary, NC). Responses against the five-point scale were dichotomized. Ratings of ‘4’ and ‘5’ were grouped and reported as positive while ratings of ‘1’, ‘2’, and ‘3’ were grouped as neutral or negative. Similarly, ratings of ‘agree’ and ‘strongly agree’ were grouped as positive while ratings of ‘neutral’, ‘disagree’, and ‘strongly disagree’ were grouped as neutral or negative. For the purpose of this article, all reporting was separated into two groups: smokers and non-smokers (those reporting to be regular or occasional smokers were considered smokers, while those reporting to be non-smokers or former smokers were collectively considered non-smokers).

Within each group, we calculated the percentages of respondents agreeing with opinion statements (Section B in the survey instrument), and the percentages of respondents reporting notice and recall of PWLs (as gauged through the first item of each of Sections D-G in the survey instrument). For each PWL and across all four outcomes of interest (salience, fear evocation, adding information, and ability to motivate quitting/staying smokefree), we calculated the percentages of respondents with positive perceptions, and compared those to the percentages obtained in 2010 in an attempt to quantify the sustained gain in perceptions. Testing for the significance of the gain was conducted using logistic regression with a significance criterion of *p* ≤ 0.05. Finally, we calculated the odds ratio for the association between the downstream outcomes of motivation and the perceptions on upstream outcomes (salience, fear evocation, and adding information) using logistic regression.

Specifically among smokers, we looked at the frequency of noticing PWLs (item C5) and associated that with considering quitting (item C8). We also looked at the recall of PWL content (item C3) and associated that with avoidance (item C7). Finally, we looked at the general intentions to quit (item C10) and associated that with the ability of each of the PWLs to motivate quitting (items 5 and 6 of sections D-G). Associations were carried out using Chi-square test.

## Results

The mean age for the sample was 20.3 years (SD = 1.6) and males (57.0%, 524/920) slightly outnumbered females (43.0%, 396/920). Of the 920 respondents considered for data analysis, 33.6% (309) were smokers while 66.4% (611) were non-smokers.

Table 1 details the proportion of smokers and non-smokers agreeing with certain statements on the harms of smoking and the need for warnings. It also details the proportion of those noticing PWLs in circulation and recalling their message content.

**Table 1:** General opinions and recall of PWLs

	Smokers (*n* = 309)	Non-smokers (*n* = 611)	*P*-value
Respondents’ opinions on the harms of smoking and the need for warnings			
*Smoking is harmful to smokers*			< 0.0001
Agree	76.7% (237)	91.7% (560)	
Neutral or disagree	23.3% (72)	8.3% (51)	
*Smoking is harmful to non-smokers*			< 0.0001
Agree	79.0% (244)	92.0% (562)	
Neutral or disagree	21.0% (65)	8.0% (49)	
*Placing warnings on cigarette packs is important*			< 0.0001
Agree	48.2% (149)	73.0% (446)	
Neutral or disagree	51.8% (160)	27.0% (165)	
Respondents noticing and recalling PWLs in circulation			
*Have previously seen*			
PWL1 (prison)	76.7% (237)	40.6% (248)	
PWL2 (child covering mouth)	80.9% (250)	53.2% (325)	
PWL3 (child using inhaler)	84.1% (260)	49.9% (305)	
PWL4 (coffin)	86.7% (268)	60.7% (371)	
*Recalled at least one statement associated with PWLs on cigarette packs*	66.3% (205)	42.1% (257)	< 0.05

Specifically among smokers, 63.1% (195/309) reported seeing PWLs frequently (every time or some of the times they held a cigarette packet). Of those, 36.4% (71/195, *p* < 0.05) reported being influenced by the PWLs enough to consider quitting and 31.8% (62/195) reported avoiding looking at them. Among smokers recalling at least one of the statements associated with PWLs on cigarette packs, 32.2% (66/205, *p* < 0.05) reported avoiding looking at them.

Among smokers who reported a general intention to quit within 6 months of survey administration (169), a significant majority (66.9%, 113/169, *p* < 0.0001) also reported PWL4 to motivate quitting. Proportions for other PWLs were lower: 18.9% for PWL1 (32/169, *p* < 0.001); 37.9% for PWL2 (64/169, *p* < 0.05); and 38.5% for PWL3 (65/169, *p* < 0.05).

**Fig. 1:**
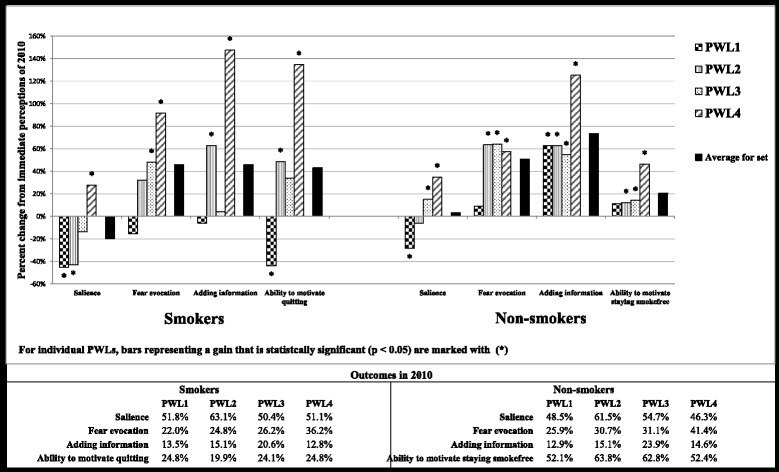
Gain in outcomes sustained in 2015

For each of the four PWLs being evaluated, and across all four outcomes of interest, Fig. 1 presents the gain sustained in 2015. For example, compared to 24.8% of smokers agreeing with the ability of PWL4 to motivate them to quit in 2010, a total of 58.2% of smokers agreed in 2015, resulting in a significant sustained gain of 134.7% ((58.2%–24.8%)/24.8%). Figure 1 also presents the gain sustained by the set of PWLs as depicted by *Average for set*, which was calculated for all outcomes of interest using the average for all four PWLs in 2015 and comparing that to the average in 2010.

For insight into the association between the gain on motivation and the gain on other upstream outcomes, Table 2 takes a closer look at PWL4 which sustained a significant positive gain in 2015 across all outcomes in both smokers and non-smokers. For example, the odds ratio for gaining on motivation given a gain on salience was 3.2 for smokers and 2.0 for non-smokers.

**Table 2:** Association between the gain on motivation and the gain on upstream outcomes of interest

	Smokers(motivation to quit)		Non-smokers (motivation to stay smokefree)	
	OR (95% CI)	*P*-value	OR (95% CI)	*P*-value
*Odds ratio for the ability of PWL4 to motivate quitting or staying smoke-free in 2015 compared to its ability in 2010*				
Respondent agrees it				
is salient	3.2 (1.7 – 5.9)	< 0.05	2.0 (1.3 – 3.1)	< 0.05
evokes fear	5.3 (2.9 – 9.6)	< 0.0001	5.0 (3.2 – 7.9)	< 0.0001
adds information	2.6 (1.4 – 4.7)	< 0.05	2.2 (1.2 – 4.0)	< 0.05

## Discussion

This paper presents the methodology for and results of the first long-term assessment of PWLs in Jordan and the EMR. To the best of our knowledge, there are no similar assessments from the EMR and only a few from LMICs, thus our findings could potentially inform decisions in other countries. Moreover, and in the absence of systematic cohort studies like the International Tobacco Control Policy Evaluation Project, [25] we believe that our methodology could provide a model for other countries to use to assess the effectiveness of PWLs.

After circulating for 2.5 years on the Jordanian market, our results (Table 1) indicate public awareness of the set of PWLs. About ¾ of smokers and ½ of non-smokers reported having seen one or more of the PWLs in circulation, and considerable proportions of both smokers (66.3%) and non-smokers (42.1%) were capable of recalling at least one of the messages associated with the PWLs.

Specifically among smokers who are aware of the PWLs, our results indicate an overall effectiveness of the set. Among those reporting frequently seeing the PWLs, 1/3 reported the PWLs to have influenced them to the extent they considered quitting. Among those reporting recalling the messages of the PWLs, 1/3 reported avoiding looking at PWLs; a quality which has been shown to be an indication of effectiveness [7, 26–28].

Overall effectiveness of the set among smokers as well as non-smokers can also be seen through examining results on the outcomes of motivation, fear evocation, and adding information (as depicted by *Average for set* in Fig. 1). With repeated exposure, the set seems to have achieved and consequently sustained some gain on its ability to motivate smokers to quit and non-smokers to stay smoke-free. Looking further upstream indicates that the set has also achieved and sustained a gain on the cognitive and emotional reactions of evoking fear and adding information.

Despite these sustained gains, the results on salience suggest that the set may have reached its end of life. Evidence concludes that the effectiveness of PWLs peaks shortly after implementation, and that salience is the first dimension to suffer erosion thereafter [5, 11, 29]. In line with this evidence, our results point in the direction of imminent wear-out of the set: the salience among smokers has dropped below the initial values obtained in 2010 (which is expected with habituation since 63.1% of smokers reported seeing them frequently), and among non-smokers the set seems to sustain only marginal salience beyond that of 2010.

At the level of individual PWLs, our results suggest variability in performance (as shown by sustained gains for individual PWLs in Fig. 1). PWL4 outperformed all others by being the only one to sustain a significant positive gain in 2015 on all outcomes including salience; a proven sign of the strength for PWLs [13]. Accordingly, our data suggest PWL4 to be stronger than others, especially when all four PWLs started at comparable levels of impact in 2010. A closer look at PWL4 (Table 2) indicates that the gain on motivation among both smokers and non-smokers (which was significantly greater than that for other PWLs) was associated with the gain on all three upstream outcomes, with fear evocation displaying the strongest association. This is in line with the evidence on the association between negative emotional reactions and intentions to quit [5, 28].

While not strictly graphic, PWL4 may have outperformed others by being along the lines of depicting disease and human suffering, a characteristic capable of eliciting emotional arousal (such as fear) which is a key driver of motivation [5, 29]. Accordingly, and since 1 in 4 smokers reports disagreement with smoking being harmful to smokers (Table 1), future PWLs should include diversified content that targets individuals at various stages of the change process [30]. This is specifically valuable since more smokers report getting information about the risks of smoking from PWLs compared to other sources [5].

Our study has its limitations. First, it focused on a sample of young educated adults and did not extend the assessment to other socio-economic groups. However, previous studies suggest that in line with research on mass media interventions, PWLs perform comparably among various subgroups [12]. Second, the study relied on cross-sectional sampling at both time points rather that employing a cohort study design. However, the sampling mechanism attempted to harmonize the study population to the extent possible.

## Conclusions

Based on the early signs of wear-out (i.e. erosion of salience), and based on understanding the importance of sustaining a gain on upstream outcomes (especially fear evocation) to sustain motivation, we recommend starting the process of retiring this set of PWLs and replacing it with a stronger one. Our recommendation is to stay in line with proven standards. For enhanced salience, the new set should have the PWLs on the front side of the packet (or on both sides), [29] and enlarge them to cover at least 50% of the surface area while minimizing the area occupied by text within the PWL [3, 5, 24]. For enhanced engagement, arousal, and effectiveness, the set should utilize gruesome images that depict disease and avoid symbolic abstract imagery [5, 29]. Based on emerging evidence, we also recommend considering ‘innovative policy configurations’ that could delay wear out such as combining PWLs with inserts or with plain packaging [31].

Although not specifically addressed within this study, the revision should tackle an important weakness of health warning policy in Jordan: the lack of any information on quitting. While motivation is critical for the actual quitting behavior, it is not enough; a smoker needs a sense of self-efficacy to trigger a quit attempt [7, 11, 20]. Accordingly, revised tobacco control policies in Jordan should consider providing smokers with information on the available tobacco dependence treatment services and on behavioral recommendations and coping skills to support unaided quitting. While such information could be included in PWLs, there is evidence of the association between reading pack inserts that carry efficacy messages and sustained quit attempts [32]. Thus, for enhanced effectiveness, we recommend considering of the use of package inserts to supplement the content of PWLs.

Our recommendation comes at a critical time when the high prevalence of smoking in Jordan is still on the rise. Only strict implementation of MPOWER strategies (six demand-specific policies the World Health Organization recommends to counter the tobacco epidemic) [33] is capable of reversing further growth in prevalence. Independent of other strategies, strong PWLs are forecasted to achieve a 3.0% drop in prevalence in the first five years (as estimated by the SimSmoke model, presentation at the Regional Meeting to Achieve the NCD Tobacco Target held by World Health Organization – EMR Office on June 8, 2015 in Tunisia).

## Additional file

Additional file 1: English translation of survey instrument. An English translation of the survey instrument used to collect data. (PDF 41 kb)

## Abbreviations

EMR: Eastern Mediterranean Region
FCTC: Framework Convention on Tobacco Control
LMICs: Low- and Middle-Income Countries
PWLs: Pictorial warning labels
