# Supplementary material for: Informing tobacco control policy in Jordan: assessing the effectiveness of pictorial warning labels on cigarette packs
Source: BMC Public Health. 2017 Aug 1;18:84. doi: 10.1186/s12889-017-4642-8 (PMC5540564; doi:10.1186/s12889-017-4642-8)
Supplement: Additional file 1: — English translation of survey instrument. An English translation of the survey instrument used to collect data. (PDF 41 kb) [file 12889_2017_4642_MOESM1_ESM.pdf]

Surveyor name: Survey serial number:

Evaluating the effectiveness of pictorial warning labels on cigarette packs

Section A:

A1	Sex	- Male<br>- Female
A2	Age	____ years
A3	Major	
A4	Year	

Section B:

**To what extent do you agree with the following statements**		Strongly disagree	Disagree	Neutral	Agree	Strongly agree
B1	Smoking is harmful to smokers					
B2	Exposure to cigarette smoke is harmful to non-smokers					
B3	Exposure to cigarette smoke is harmful to smokers					
B4	It is important to have health warnings on cigarette packs					

Section C:

Please choose the most appropriate response		
C1	In the last month, did you notice any health warning on cigarette packs?	- Yes<br>- No ***(skip to C4)***<br>- I have not seen any cigarette packs ***(skip to C4)***
C2	Where is it located?	- Front<br>- Side<br>- Back<br>- Both front and back
C3	What does it say? You can choose more than one statement	- Smoking is an addition, do not start<br>- Smoking affects children, protect your children from smoking<br>- Keep your children away from smokers to protect them from respiratory diseases<br>- Smoking causes slow and painful death<br>- Other (specify......)<br>- I don't know
C4	Currently you are	- A regular cigarette smoker, _____ cigs/day<br>- An occasional cigarette smoker<br>- Former smoker ***(skip to D1)***<br>- Non-smoker ***(skip to D1)***
C5	In the last month, how many times did you notice the health warning on cigarette packs?	- Every time I held a cigarette pack<br>- Some of the times I held a cigarette pack<br>- Rarely<br>- I never noticed it
C6	Do you feel any unease due to the presence of health warnings on cigarette packs?	- No<br>- Yes
C7	In the last month, did you avoid looking at health warnings on cigarette packs (for example through covering them)?	- No<br>- Yes
C8	In the last month, did any health warning on cigarette packs affect you to the extent you are considering quitting?	- No<br>- Yes
C9	In your opinion, health warnings on cigarette packs should include _______ information on health	- Less<br>- More<br>- Same amount of
C10	Are you thinking about quitting in the coming 6 months?	- No<br>- Yes
C11	Did you try quitting in the past 12 months?	- No ***(skip to D1)***<br>- Yes
C12	When was this attempt?	- Less than 6 months ago<br>- More than 6 months ago

Section D: Warning 1 (show image):

D1	Have you previously seen this warning?	- No<br>- Yes

**Please circle the number from 1 to 5 that best represents your perception of the warning that you have just seen.**			
D2	Can be easily ignored	1 2 3 4 5	Attracts attention
D3	Not fearful	1 2 3 4 5	Very fearful
D4	Did not add to my information	1 2 3 4 5	Added to my information

**If you are a non-smoker, please complete this section.**		Strongly disagree	Disagree	Neutral	Agree	Strongly agree
D5	Seeing this warning motivates to stay smoke-free					

**If you are a smoker, please complete this section.**		Strongly disagree	Disagree	Neutral	Agree	Strongly agree
D6	Seeing this warning motivates to quit smoking					

Section E: Warning 2 (show image):

E1	Have you previously seen this warning?	- No<br>- Yes

**Please circle the number from 1 to 5 that best represents your perception of the warning that you have just seen.**			
E2	Can be easily ignored	1 2 3 4 5	Attracts attention
E3	Not fearful	1 2 3 4 5	Very fearful
E4	Did not add to my information	1 2 3 4 5	Added to my information

**If you are a non-smoker, please complete this section.**		Strongly disagree	Disagree	Neutral	Agree	Strongly agree
E5	Seeing this warning motivates to stay smoke-free					

**If you are a smoker, please complete this section.**		Strongly disagree	Disagree	Neutral	Agree	Strongly agree
E6	Seeing this warning motivates to quit smoking					

Section F: Warning 3 (show image):

F1	Have you previously seen this warning?	- No<br>- Yes

**Please circle the number from 1 to 5 that best represents your perception of the warning that you have just seen.**			
F2	Can be easily ignored	1 2 3 4 5	Attracts attention
F3	Not fearful	1 2 3 4 5	Very fearful
F4	Did not add to my information	1 2 3 4 5	Added to my information

**If you are a non-smoker, please complete this section.**		Strongly disagree	Disagree	Neutral	Agree	Strongly agree
F5	Seeing this warning motivates to stay smoke-free					

**If you are a smoker, please complete this section.**		Strongly disagree	Disagree	Neutral	Agree	Strongly agree
F6	Seeing this warning motivates to quit smoking					

Section G: Warning 4 (show image):

G1	Have you previously seen this warning?	- No<br>- Yes

**Please circle the number from 1 to 5 that best represents your perception of the warning that you have just seen.**			
G2	Can be easily ignored	1 2 3 4 5	Attracts attention
G3	Not fearful	1 2 3 4 5	Very fearful
G4	Did not add to my information	1 2 3 4 5	Added to my information

**If you are a non-smoker, please complete this section.**		Strongly disagree	Disagree	Neutral	Agree	Strongly agree
G5	Seeing this warning motivates to stay smoke-free					

**If you are a smoker, please complete this section.**		Strongly disagree	Disagree	Neutral	Agree	Strongly agree
G6	Seeing this warning motivates to quit smoking					
